# Endothelium‐Derived Engineered Extracellular Vesicles Protect the Pulmonary Endothelial Barrier in Acute Lung Injury

**DOI:** 10.1002/advs.202306156

**Published:** 2023-12-07

**Authors:** Zhengyan Gu, Mingxue Sun, Jihao Liu, Qi Huang, Yunqin Wang, Jun Liao, Tingbin Shu, Min Tao, Guanchao Mao, Zhipeng Pei, Wenqi Meng, Xinkang Zhang, Youheng Wei, Shanshan Zhang, Songling Li, Kai Xiao, Ying Lu, Qingqiang Xu

**Affiliations:** ^1^ Lab of Toxicology and Pharmacology Faculty of Naval Medicine Naval Medical University Shanghai 200433 P. R. China; ^2^ Department of Pharmaceutical Sciences School of Pharmacy Naval Medical University Shanghai 200433 P. R. China; ^3^ School of Traditional Chinese Materia Medica Shenyang Pharmaceutical University Shenyang 110006 P. R. China; ^4^ School of Medicine Shanghai University Shanghai 200444 P. R. China; ^5^ State Key Laboratory of Genetic Engineering Institute of Genetics Fudan University Shanghai 200433 P. R. China; ^6^ Marine Biomedical Science and Technology Innovation Platform of Lingang Special Area Shanghai 200433 P. R. China; ^7^ Basic Medical Center for Pulmonary Disease Faculty of Naval Medicine Naval Medical University Shanghai 200433 P. R. China

**Keywords:** acute lung injury, EGR1, engineered extracellular vesicles, extracellular vesicles, pulmonary endothelial barrier

## Abstract

Acute lung injury (ALI) is a severe respiratory disease with a high mortality rate. The integrity of the pulmonary endothelial barrier influences the development and prognosis of ALI. Therefore, it has become an important target for ALI treatment. Extracellular vesicles (EVs) are promising nanotherapeutic agents against ALI. Herein, endothelium‐derived engineered extracellular vesicles (eEVs) that deliver microRNA‐125b‐5p (miRNA‐125b) to lung tissues exerting a protective effect on endothelial barrier integrity are reported. eEVs that are modified with lung microvascular endothelial cell‐targeting peptides (LET) exhibit a prolonged retention time in lung tissues and targeted lung microvascular endothelial cells in vivo and in vitro. To improve the efficacy of the EVs, miRNA‐125b is loaded into EVs. Finally, LET‐EVs‐miRNA‐125b is constructed. The results show that compared to the EVs, miRNA‐125b, and EVs‐miRNA‐125b, LET‐EVs‐miRNA‐125b exhibit the most significant treatment efficacy in ALI. Moreover, LET‐EVs‐miRNA‐125b is found to have an important protective effect on endothelial barrier integrity by inhibiting cell apoptosis, promoting angiogenesis, and protecting intercellular junctions. Sequencing analysis reveals that LET‐EVs‐miRNA‐125b downregulates early growth response‐1 (EGR1) levels, which may be a potential mechanism of action. Taken together, these findings suggest that LET‐EVs‐miRNA‐125b can treat ALI by protecting the endothelial barrier integrity.

## Graphical abstract

Endothelium‐derived engineered extracellular vesicles (LET‐EVs‐miRNA‐125b) are established for the first time, which can target lung tissue and enhance retention. LET‐EVs‐miRNA‐125b can inhibit cell apoptosis, restore cell junctions, and promote proliferation by delivering miRNA‐125b to endothelial cells, which has great potential in the treatment of acute lung injury.

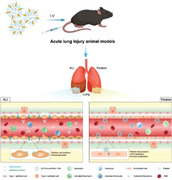

## 1 Introduction

Acute lung injury is a severe clinical syndrome caused by direct or indirect damage to lung tissues (as a result of lung infection, toxic gases, radiation, etc.).^[^ ^1^ ^]^ Inflammatory cascades and the destruction of the air‐blood barrier are the dominant pathological features.^[^ ^2^ ^]^ Without early intervention, ALI can develop into acute respiratory distress syndrome (ARDS), which has high morbidity and mortality rates. In a clinical survey, the incidence of ARDS accounted for 10.4% of ICU patients, and the in‐hospital mortality rate was 34.9–46.1%.^[^ ^3^ ^]^ Patients who survived had exercise limitations and poor quality of life.^[^ ^1b^ ^]^ Infection control, mechanical ventilation, and glucocorticoid medications are the main clinical treatments for ARDS; however, none of them significantly reduce morbidity or mortality rates.^[^ ^4^ ^]^ Changes in the structure and function of the pulmonary air‐blood barrier are a major pathogenic characteristic of ALI.^[^ ^5^ ^]^ Disruption of endothelial barrier integrity accelerates the development of ALI and affects healing after injury,^[^ ^6^ ^]^ therefore, protection of endothelial barrier integrity is a valuable strategy for the treatment of ALI.

Extracellular vesicles (EVs) are a class of small membranous vesicles secreted by cells, which play an important role in mediating intercellular communication.^[^ ^7^ ^]^ EVs carry a variety of molecules such as lipids, multiple species of RNA, various proteins, and even organelles. EVs can play a remarkable role in various diseases by transferring their contents from vesicles to recipient cells^[^ ^8^ ^]^; therefore, EVs have become an effective alternative to cell therapy.^[^ ^9^ ^]^ Recently, many studies have reported that EVs have great potential in the treatment of ALI.^[^ ^4^, ^10^ ^]^ Our preliminary research showed that mesenchymal stem cell (MSC)‐derived EVs could reduce mortality and mitigate lung injury in mice with sulfur mustard‐induced ALI.^[^ ^1a^ ^]^ However, there are some limitations to the use of native EVs for the treatment of ALI. First, native EVs have a short circulation time in vivo.^[^ ^11^ ^]^ After intravenous administration, EVs rapidly accumulate in the liver prior to metabolism; therefore, EVs cannot exert long‐lasting effects on damaged lung tissue. Second, native EVs contain low amounts of effective substances, which cannot achieve significant therapeutic effects.^[^ ^12^ ^]^

To improve their efficiency, specificity, and safety, the concept of engineered EVs (eEVs) has been proposed. The surface of EVs can be chemically or biologically modified to alter their distribution and enhance their targeting ability.^[^ ^13^ ^]^ EVs can also serve as nano‐vectors that carry therapeutic molecules (such as nucleic acids, proteins, and small chemicals).^[^ ^14^ ^]^ Recently, the use of eEVs for the treatment in ALI has developed rapidly and achieved excellent therapeutic efficacy. Salazar‐Puerta et al.^[^ ^15^ ^]^ constructed surfactant protein A (SPA) functionalized IL‐4‐ and IL‐10‐loaded eEVs to treat ALI. These eEVs exhibited significant anti‐inflammatory ability in vivo and in vitro by inhibiting macrophage activation, decreasing the secretion of pro‐inflammatory factors, and reducing neutrophil infiltration. Yohan Han et al.^[^ ^16^ ^]^ constructed eEVs loaded with the anti‐inflammatory CC16 protein for the treatment of ALI. sEV‐CC16 had excellent anti‐inflammatory activity, protecting mice from lipopolysaccharides (LPS) or bacterial‐induced ALI. Currently, eEVs have mainly been designed to inhibit inflammation in ALI, but few eEVs have been designed to protect the air‐blood barrier, the dysfunction of which plays a crucial role in the development of ALI. Here, we designed endothelium‐derived eEVs for repairing the endothelial barrier.

A lung endothelial cell‐targeted peptide (LET‐CGSPGWVRC), which was obtained by phage display technology, was modified onto the surface of endothelium‐derived EVs to achieve targeted lung delivery.^[^ ^17^ ^]^ Next, miRNA‐125b, which has been proven to have an important protective effect against ALI, was loaded into the EVs to improve the efficiency.^[^ ^18^ ^]^ Thus, eEVs‐LET‐EVs‐miRNA‐125b was obtained.

In this study, we found that LET‐EVs‐miRNA‐125b had a long residence time in vivo and specifically targeted lung microvascular endothelial cells in vivo and in vitro. In LPS‐induced ALI mice, the LET‐EVs‐miRNA125b group showed the best efficacy compared to that of the EVs, miRNA‐125b, and EVs‐miRNA‐125b treatment groups, with significantly reduced lung injury, attenuation of the inflammatory response, and protection of the air‐blood barrier. Moreover, LET‐EVs‐miRNA‐125b exerted a protective effect on endothelial barrier integrity by inhibiting endothelial cell apoptosis, promoting angiogenesis, and protecting intercellular junctions. The results of the sequencing analysis showed that LET‐EVs‐miRNA‐125b delivery of miRNA‐125b could downregulate the expression of EGR1, which is a potential mechanism for regulating inflammation and barrier repair. In conclusion, we designed and prepared LET‐EVs‐miRNA‐125b, which could be a potential therapeutic agent for ALI by protecting the integrity of the pulmonary endothelial barrier.

## 2 Results

### 2.1 Preparation and Characterization of LET‐EVs‐miRNA‐125b

**Figure 1:**
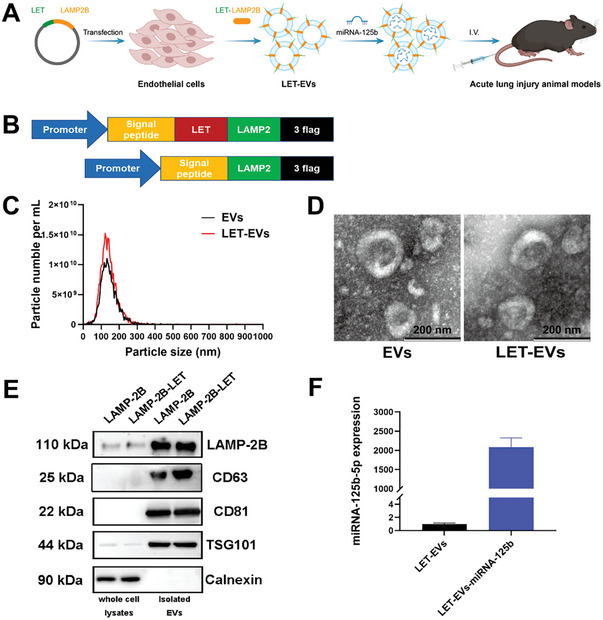
Design and characterization of LET‐EVs‐miRNA‐125b. A) Schematic diagram of the preparation of LET‐EVs‐miRNA125b. LET was modified on the surface by donor cell modification and miRNA‐125b was loaded into EVs by transfection. B) Schematics of the construction and production of the LAMP‐2B‐LET fusion protein plasmid. C) Size distribution and particles of EVs and LET‐EVs, measured using NTA. The average sizes of EVs and LET‐EVs were 135.8 and 133.6 nm, respectively. D) The morphology of EVs and LET‐EVs was a typical cup‐shaped structure detected by TEM (Scale bar: 200 nm). E) EV‐positive (LAMP‐2B, CD63, CD81, and TSG101) and ‐negative (Calnexin) markers were detected in LAMP‐2B‐EVs, LAMP‐2B‐LET‐EVs, and their donor cells by western blotting. F) The loading efficiency of miRNA‐125b‐5p was detected using qRT‐PCR to compare the relative expression of miRNA‐125b‐5p between LET‐EVs and LET‐EVs‐miRNA‐125b (*n* = 3).

To prepare LET‐EVs‐miRNA‐125b, human umbilical vein endothelial cells (HUVECs) were transfected with lentivirus encoding lysosome‐associated membrane glycoprotein 2b (LAMP‐2B) or LAMP‐2B‐LET (**Figure** **1**A,B). The expression of green fluorescent protein confirmed that the lentiviruses were successfully expressed in HUVECs (Figure S1A, Supporting Information). In contrast to non‐transfected HUVECs, the mRNA and protein expressions of LAMP‐2B were evidently upregulated in LAMP‐2B‐LET and LAMP‐2B transfected HUVECs (Figure S1B,C, Supporting Information). Secreted LET‐EVs and EVs were isolated using size‐exclusion chromatography. Nanoparticle tracking analysis (NTA) showed that the average sizes of EVs and LET‐EVs were 135.8 nm and 133.6 nm and the particle numbers of EVs and LET‐EVs were 2.0 × 10^11^ and 2.6 × 10^11^ particles per milliliter of PBS, respectively (Figure 1C). The morphology of EVs under transmission electron microscopy（TEM） revealed that both EVs and LET‐EVs had a typical cup‐shaped structure of less than 200 nm (Figure 1D). Western blotting results demonstrated that, in contrast to the whole cell lysates, purified EVs showed enrichment of EV markers (LAMP‐2B, CD81, CD63, and TSG101), but were negative for the calnexin marker, which further validated the successful isolation of EVs (Figure 1E). miRNA‐125b was then loaded into LET‐EVs and LET‐EVs‐miRNA‐125b was purified. The relative expression of miRNA‐125b in LET‐EVs‐miRNA‐125b was elevated by almost 2000 times compared to that in the LET‐EVs (Figure 1F). To validate the capacity of LET‐EVs for the delivery of miRNA‐125b to endothelial cells, miRNA‐125b and LET‐EVs were fluorescently labeled and co‐cultured with HUVECs for 2 h. Fluorescence microscopy results showed that Cy5‐labeled miRNA‐125b co‐localized with DIO‐labeled LET‐EVs in HUVECs, indicating that LET‐EVs‐miRNA‐125b could deliver miRNA125b into HUVECs (Figure S2, Supporting Information).

### 2.2 Uptake and Distribution of LET‐EVs‐miRNA‐125b In Vitro and In Vivo

**Figure 2:**
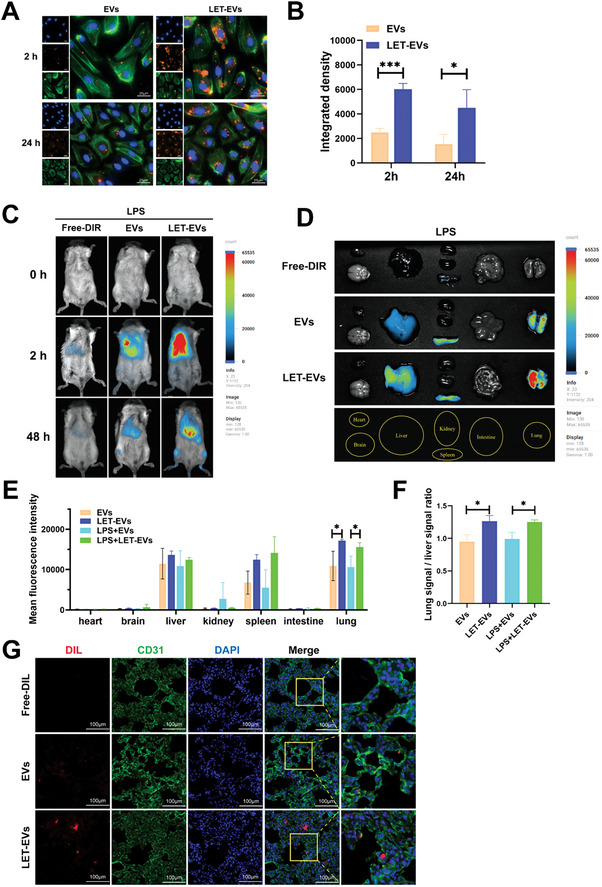
Uptake and distribution of LET‐EVs and EVs in vivo and in vitro. A) Fluorescence microscope of cellular uptake of DIL‐labeled LET‐EVs and EVs after 2 and 24 h of incubation with HPMVECs. The stains used were as follows: DIL‐labeled EVs (Orange), F‐actin (Green), and DAPI (Blue) (Scale bar: 25 µm). B) Quantification of EVs integrated fluorescence density based on ImageJ analysis. The uptake efficiency of DIL‐labeled LET‐EVs was higher than that of DIL‐EVs. C) Imaging of ALI mice after 0, 2, and 48 h of administration of DIR, DIR‐EVs, and DIR‐LET‐EVs. Compared with the DIR and DIR‐EVs groups, the fluorescence signal of LET‐EVs was mainly accumulated in the epigastric region after 2 h. D) Fluorescence imaging of tissues from ALI mice in the DIR, DIR‐EVs, and DIR‐LET‐EVs groups after 48 h. Compared with the DIR and DIR‐EVs groups, the signal in the DIR‐LET‐EVs groups was mainly aggregated in lung tissues. E) Quantitative analysis of mean fluorescence intensities in different tissues following administration of EVs and LET‐EVs. F) The clearance of EVs in lung tissues was calculated by dividing average lung signals by average liver signals. G) Uptake of DIL‐EVs and DIL‐LET‐EVs by endothelial cells in lung tissues. Immunofluorescence staining was performed in lung sections using an antibody against CD31 (endothelial cell marker, green). Nuclei were stained with DAPI (Scale bar: 100 µm). All the data are presented as the mean ± SD (*n* = 3). ^*^ *p* < 0.05, ^***^ *p* < 0.001 compared with the EVs group by unpaired Student's *t*‐tests.

To assess the targeting ability of LET‐EVs, we labeled EVs and LET‐EVs with the membrane dye DIL (shown in orange color). Human pulmonary microvascular endothelial cells (HPMVECs) were incubated with DIL‐labeled EVs and LET‐EVs at 37 °C for 2 and 24 h. Fluorescence microscopy results showed that the uptake efficiency of LET‐EVs in HPMVECs was 2‐fold higher than that of EVs incubated for 2 h. After 24 h, the uptake efficiencies of both LET‐EVs and EVs decreased, but the uptake efficiency of LET‐EVs was still higher than that of EVs (**Figure** **2**A,B). We then investigated the distribution of LET‐EVs in vivo. Healthy and ALI mice received the same number of DIR‐labeled LET‐EVs and EVs via intravenous injection, followed by fluorescence imaging at 0, 2, and 48 h. To avoid a false‐positive DIR signal, the same labeling step was performed with a group that did not receive EVs to serve as a negative control. As shown in Figure 2C and Figure S3A (Supporting Information), the fluorescence signal of LET‐EVs was mainly accumulated in the epigastric region after 2 h, which differed from that of EVs. After 48 h, the signal decreased, but LET‐EVs showed a longer residence time in vivo compared to that of EVs (Figure 2C; Figure S3A, Supporting Information). Mice were euthanized and major tissues were collected for ex vivo imaging after 48 h. The mean fluorescence signal from all tissues was used to compare the distribution of LET‐EVs in mice. The results showed that LET‐EVs exhibited a higher fluorescence signal in lung tissues than that of EVs in both healthy and ALI mice (Figure 2D,E; Figure S3B, Supporting Information). The clearance of EVs in lung tissues was calculated by dividing average lung signals by average liver signals. There was a statistically significant difference between the clearance of LET‐EVs and EVs in lung tissues, suggesting LET‐EVs exhibit a prolonged retention time in lung tissues and a reduced rate of removal (Figure 2F). To determine whether endothelial cells interact with LET‐EVs in lung tissues, DIL‐labeled LET‐EVs and EVs were intravenously administered to mice with ALI. Lung tissues were stained with anti‐CD31 (green) to visualize the endothelial cells. The results showed that LET‐EVs co‐localized with CD31‐positive cells, suggesting that LET‐EVs have a lung endothelial cell targeting ability (Figure 2G).

### 2.3 LET‐EVs‐miRNA‐125b Ameliorates ALI in Mice

To assess the therapeutic effect of LET‐EVs‐miRNA‐125b, we first established an animal model of ALI by performing tracheal nebulization with 1 mg kg^−1^ LPS. Mice were euthanized after 4 and 48 h. The levels of inflammation and lung injury in control and model mice were detected to evaluate the reliability of the ALI animal models (Figure S4, Supporting Information). Hematoxylin and eosin (H&E) staining and lung tissue morphometric analysis were used to assess lung injury. The lung injury score was calculated by the lung injury scoring system (Table S2, Supporting Information). Compared with that of the control group, the morphology of the lung tissues was slightly changed after 4 h and significantly changed after 48 h in ALI mice, with a decrease in the number of alveoli per high power field (hpf) and increased alveolar septal thickness and mean linear intercept, resulting in severe lung injury (Figure S4A,B, Supporting Information). Protein concentration in bronchoalveolar lavage fluid (BALF) is an important indicator for evaluating the integrity of the air‐blood barrier. The results showed that the protein concentration increased after LPS stimulation for 4 and 48 h in ALI mice, compared with that of the control group, which indicated that the air‐blood barrier had been disrupted (Figure S4D, Supporting Information). Inflammatory cells and cytokine levels in BALF were measured to evaluate the inflammatory response induced by LPS. The results of Wright‐Giemsa staining and the inflammatory cell count showed that a large number of neutrophils were aggregated in the alveoli/interstitium after 4 h of stimulation, and the number of neutrophils gradually increased after 48 h (Figure S4C, Supporting Information). The concentration of Interleukin‐6 (IL‐6), IL‐1β, and tumor necrosis factor‐alpha (TNF‐α) was also increased in BALF compared to that of the control group (Figure S4E, Supporting Information). These results confirmed that our ALI animal model was successfully established.

**Figure 3:**
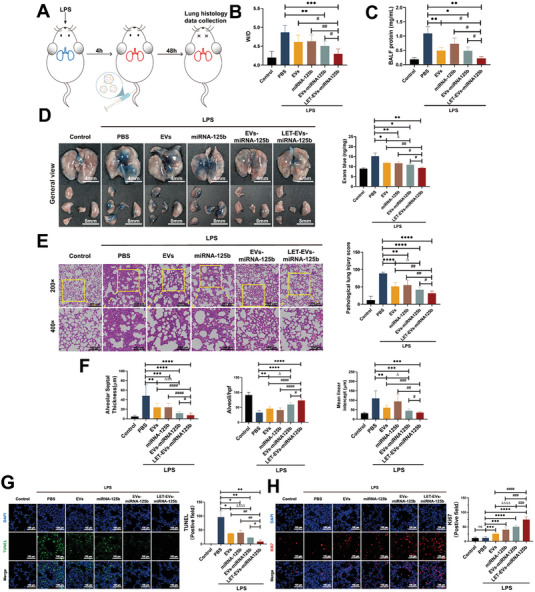
LET‐EVs‐miRNA‐125b ameliorates ALI in mice. A) Schematic diagram depicting the timeline of intratracheal administration of 1 mg kg^−1^ LPS, treatment with LET‐EVs‐miRNA‐125b after 4 h, and subsequent analysis of lung injury parameters after 48 h. B) Wet‐to‐Dry weight ratio of lung tissues. C) Total protein concentration in BALF. D) Representative images of lung tissues after staining with Evans blue. The concentration of Evans Blue in lung tissues was detected using a microplate reader. W/D ratio, BALF protein concentration, and the concentration of Evans blue in lung tissues were decreased after treatment with LET‐EVs‐miRNA‐125b compared to those in the PBS group. E) Representative images of H&E‐stained lung tissue sections. The pathological lung injury score was calculated according to the H&E‐staining. F) The morphology of the lung tissues showed an increase in the number of alveoli/hpf and decreased alveolar septal thickness and mean linear intercept after treatment with LET‐EVs‐miRNA‐125b, compared to that in the PBS group. G,H) TUNEL staining of apoptotic cells and KI67 staining of proliferating cells in lung tissue sections (Scale bar: 100 µm). ImageJ was used to quantify fluorescence intensity. Many cells in the PBS group were apoptotic and a few cells were proliferating. After treatment with LET‐EVs‐miRNA‐125b, a large number of cells were proliferating and cell apoptosis was inhibited. All data are presented as the mean ± SD (*n* = 3). ^*^ *p* < 0.05, ^**^ *p* < 0.01, ^***^ *p* < 0.001, ^****^ *p* < 0.0001, compared with the PBS treatment group by unpaired Student's *t*‐tests. ^#^ *p* < 0.05, ^##^ *p* < 0.01, ^###^ *p* < 0.001, ^####^ *p* < 0.0001, compared with the LET‐EVs‐miRNA‐125b treatment group by unpaired Student's *t*‐tests. ^∆^ *p* < 0.05, ^∆∆∆∆^ *p* < 0.0001 of the EVs treatment group compared with the EVs‐miRNA‐125b treatment group by unpaired Student's *t*‐tests.

**Figure 4:**
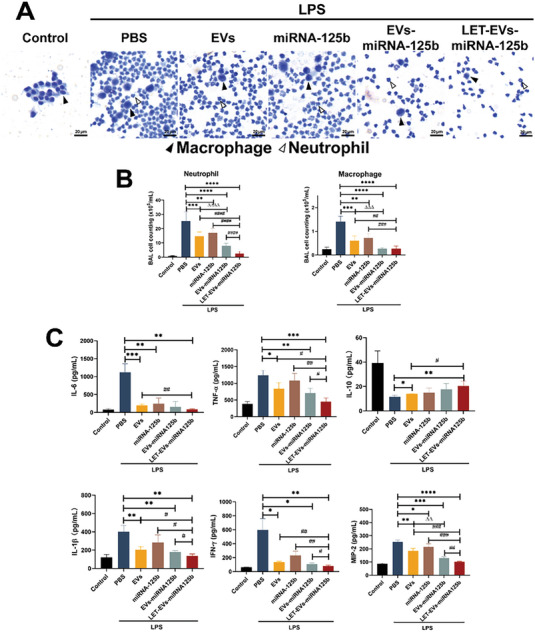
LET‐EVs‐miRNA‐125b inhibits inflammatory response in ALI mice. A) Representative images of Wright‐Giemsa staining of BALF cells in different groups (Scale bar: 100 µm). B) The numbers of macrophages and neutrophils in BALF. After LPS stimulation, a large number of neutrophils and macrophages were aggregated in the alveoli/interstitium and the recruitment was reduced in the LET‐EVs‐miRNA‐125b treatment group. C) The concentration of IL‐6, IL‐10, TNF‐α, IL‐1β, MIP‐2, and IFN‐γ in BALF detected by Elisa.

We further evaluated the efficacy of LET‐EVs‐miRNA‐125b in ALI. After LPS stimulation for 4 h, mice received 1 × 10^10^ LET‐EVs‐miRNA‐125b by intravenous injection and were then euthanized after 48 h (**Figure** **3**A). H&E staining and morphometric analysis showed that, compared to that of the PBS group, lung injury could be alleviated by treatment with LET‐EVs‐miRNA‐125b due to an increase in the number of alveoli/hpf and a decrease in the alveolar septal thickness and the mean linear intercept (Figure 3E,F). The wet‐to‐dry weight (W/D) and BALF protein levels, which are important indicators of pulmonary edema, were significantly decreased in lung tissues following administration of LET‐EVs‐miRNA‐125b (Figure 3B,C). The pulmonary vascular leakage experiment showed that LET‐EVs‐miRNA‐125b could reduce the dispersion of Evans Blue in the lung tissues, due to reduced vascular permeability (Figure 3D). Moreover, LET‐EVs‐miRNA‐125b showed remarkable anti‐inflammatory activities. Wright‐Giemsa staining and inflammatory cell count showed that neutrophil recruitment was reduced in the LET‐EVs‐miRNA‐125b treatment group (**Figure** **4**A,B). The expression of pro‐inflammatory cytokine and chemotaxis (IL‐6, IL‐1β, TNF‐α, Macrophage inflammatory protein 2 (MIP‐2), and Interferon γ(IFN‐γ) was decreased and anti‐inflammatory cytokine expression (IL‐10) was increased in BALF after administration of LET‐EVs‐miRNA‐125b, compared to those in the PBS group (Figure 4C). The pulmonary function indices of the respiratory rate showed that the respiratory rate was increased in ALI mice and was decreased to a certain extent after treatment with LET‐EVs‐miRNA‐125b (Figure S5, Supporting Information).

To further explore the protective effect of LET‐EVs‐miRNA‐125b against ALI, we examined cell proliferation and apoptosis. The results showed that the densities of KI67‐positive cells (proliferative) were not significantly different between the PBS and control groups. After treatment with LET‐EVs‐miRNA‐125b, the density of KI67‐positive cells significantly increased. In addition, the numerical density of TUNEL‐positive cells was significantly increased in the PBS group compared to that in the control group but decreased after administration of LET‐EVs‐miRNA‐125b (Figure 3G,H). Compared with the EVs, miRNA‐125b, and EVs‐miRNA‐125b groups, LET‐EVs‐miRNA‐125b showed the best treatment efficacy in ALI mice.

### 2.4 LET‐EVs‐miRNA‐125b Protects the Integrity of the Endothelial Barrier

**Figure 5:**
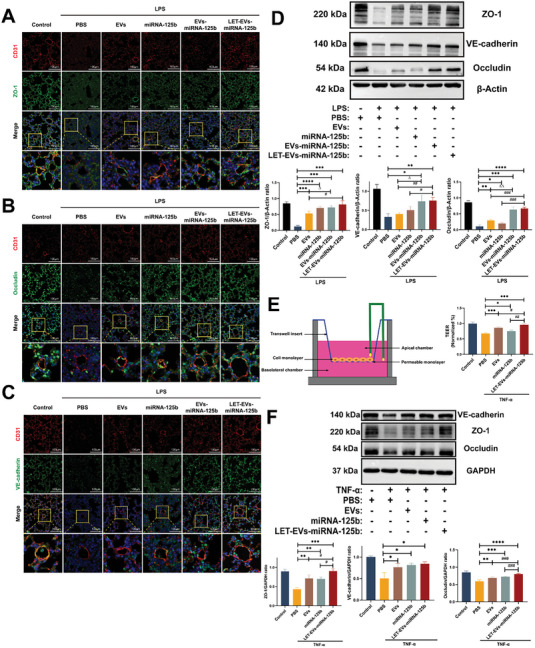
The distribution and expression of junction proteins in lung tissues and cell models after treatment with LET‐EVs‐miRNA‐125b. A–C) Immunofluorescence staining showing ZO‐1 (Green), Occludin (Green), and VE‐cadherin (Green) expression in lung tissues. CD31 (Red) was used to label endothelial cells. Nuclei were stained with DAPI (Blue) (Scale bar: 100 µm). The fluorescence intensity was low in the PBS group and exhibited a fragmented staining pattern. After treatment with LET‐EVs‐miRNA‐125b, the fluorescence intensity was significantly higher with continuous membrane staining. D) ZO‐1, Occludin, and VE‐cadherin expression in lung tissues in different groups, detected by western blotting. The levels of ZO‐1, Occludin, and VE‐cadherin were quantitated using ImageJ and normalized to β‐Actin. The expressions of ZO‐1, Occludin, and VE‐cadherin in the PBS group were significantly decreased but were restored to different degrees after treatment with EVs, miRNA‐125b, EVs‐miRNA‐125b, and LET‐EVs‐miRNA‐125b. E) Schematic diagram depicting the establishment of an endothelial barrier injury cell model. HUVECs were seeded in Transwell filter inserts to form a cell monolayer. TEER values were measured to assess the integrity of the endothelial barrier. The data were normalized to the control group. F) ZO‐1, Occludin, and VE‐cadherin expression in endothelial cells in different groups detected using western blotting. The levels of ZO‐1, Occludin, and VE‐cadherin were quantitated using ImageJ and normalized to GAPDH. All data are presented as the mean ± SD (*n* = 3). ^*^ *p* < 0.05, ^**^ *p* < 0.01, ^***^ *p* < 0.001, ^****^ *p* < 0.0001, compared with the PBS treatment group by unpaired Student's *t*‐tests. ^#^ *p* < 0.05, ^##^ *p* < 0.01, ^###^ *p* < 0.001, compared with the LET‐EVs‐miRNA‐125b treatment group by unpaired Student's *t*‐tests. ^∆^ *p* < 0.05, ^∆∆^ *p* < 0.01, the EVs treatment group compared with the EVs‐miRNA‐125b treatment group by unpaired Student's *t*‐tests.

After LET‐EVs‐miRNA‐125b treatment, vascular permeability was significantly reduced in ALI mice, suggesting that LET‐EVs‐miRNA‐125b may have an important protective effect on the endothelial barrier. ZO‐1, Occludin, and VE‐cadherin are the most important junction proteins in endothelial cells that maintain endothelial barrier integrity. Immunofluorescence (IF) staining and immunoblotting (IB) were performed to study the distribution and expression of junction proteins in lung tissues. Lung tissues were stained with an anti‐CD31 antibody (red) to visualize endothelial cells. The results showed that ZO‐1 in CD31 positive cells had a high fluorescence intensity and a continuous line along the boundaries in the control group. However, after treatment with LPS, the fluorescence intensity decreased significantly, and a fragmented staining pattern was observed. After treatment with EVs and miRNA‐125b, the immunofluorescence intensity was elevated, and partial cell membrane staining was restored. After treatment with LET‐EVs‐miRNA‐125b, the fluorescence intensity was significantly higher with continuous membrane staining. The immunostaining properties of Occludin and VE‐cadherin were similar to those of ZO‐1 (**Figure** **5**A–C). Western blotting results showed that the expression of ZO‐1, Occludin, and VE‐cadherin in the LPS treatment group was significantly decreased but was restored to different degrees after treatment with EVs, miRNA‐125b, EVs‐miRNA‐125b, and LET‐EVs‐miRNA‐125b. The LET‐EVs‐miRNA‐125b treatment group showed the highest expression of junction proteins compared to that of the other treatment groups (Figure 5D). However, the cellular composition of lung tissues is complex; therefore, in order to further verify the mechanism of LET‐EVs‐miRNA125b action in endothelial cells, an endothelial barrier injury cell model was established to further validate the protective effect of LET‐EVs‐miRNA‐125b at the cellular level. TNF‐α is an important pro‐apoptotic cytokine in the pathogenesis of ALI, which directly damages HPMVECs and induces endothelial barrier disruption.^[^ ^19^ ^]^ Therefore, TNF‐α was used to damage the endothelial barrier in the cell model. The cell monolayer of HUVECs was pre‐treated with TNF‐α (10 ng/mL) for 4 h and then treated with PBS, EVs, miRNA‐125b, and LET‐EVs‐miRNA125b. After 24 h, transendothelial electrical resistance (TEER) values were measured using a Voltohmmeter. Compared to those of the control group, TEER values were significantly decreased in the TNF‐α treated group. After treatment with LET‐EVs‐miRNA‐125b, the values increased by 25%, which indicates that LET‐EVs‐miRNA‐125b protected the integrity of the endothelial barrier (Figure 5E). Then, the distribution and expression of junction proteins in the endothelial cells were studied using IF and IB. These results were similar to those observed in lung tissues (Figure 5F; Figure S7, Supporting Information).

### 2.5 The Protective Effect of LET‐EVs‐miRNA‐125b on Injured Endothelial Cells

**Figure 6:**
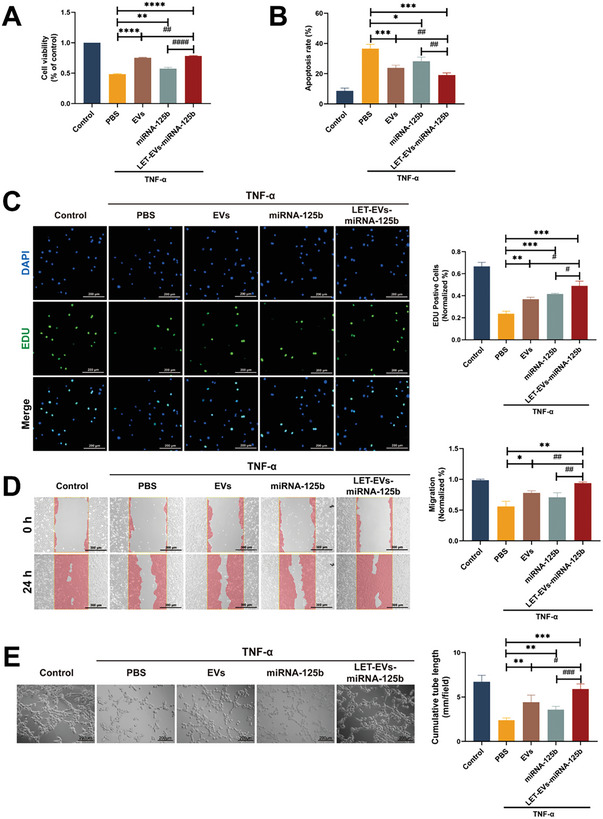
The protective effect of LET‐EVs‐miRNA‐125b on injured endothelial cells. A) Cell viability was measured using CCK‐8 assays. B) The quantity of apoptotic cells is presented as the percent of total cells. C) Cell proliferation was measured using EDU assays. EdU positive cells (Green) and Hoechst positive cells (Blue) are shown (Scale bar: 200 µm). D) A scratch experiment was used to assess cell migration (Scale bar: 200 µm). E) Representative images of HUVEC tube formation (Scale bar: 200 µm). The cumulative tube length was used to assess the angiogenesis ability in each group. All data are presented as the mean ± SD (*n* = 3). ^*^ *p* < 0.05, ^**^ *p* < 0.01, ^***^ *p* < 0.001, ^****^ *p* < 0.0001, compared with the PBS treatment group by unpaired Student's *t*‐tests. ^#^ *p* < 0.05, ^##^ *p* < 0.01, ^###^ *p* < 0.001, ^####^ *p* < 0.0001, compared with the LET‐EVs‐miRNA‐125b treatment group by unpaired Student's *t*‐tests.

To further explore the protective effect of LET‐EVs‐miRNA‐125b on injured endothelial cells in ALI, cell viability, apoptosis, proliferation, and angiogenesis were examined in an injured endothelial cell model. TNF‐α was used to stimulate HUVECs to establish the injured endothelial cell model. In the CCK‐8 experiment, the results showed that the viability of HUVECs significantly decreased after TNF‐α stimulation but recovered after administration of LET‐EVs‐miRNA‐125b for 24 h (**Figure** **6**A). We then investigated the role of LET‐EV‐miRNA‐125b in apoptosis. TNF‐α induced apoptosis in a large number of HUVECs (36.64%), while in the EVs and miRNA‐125b groups, the level of apoptosis decreased by 23.85% and 28.22%, respectively. The LET‐EVs‐miRNA‐125b group exhibited the best therapeutic effect and the rate of apoptosis was 19.18%, which was statistically significantly different compared to that of the TNF‐α group (*P*<0.001) (Figure 6B; Figure S8, Supporting Information). The EdU proliferation assay was used to evaluate the ability of LET‐EVs‐miRNA‐125b to repair damaged endothelial cells. The results showed that injured HUVECs had a poor proliferative ability. When EVs, miRNA‐125b, and LET‐EVs‐miRNA‐125b were administered, cell proliferation increased significantly. In the EDU proliferation assay, miRNA‐125b had a better effect on promoting proliferation than EVs (Figure 6C). The migration experiment showed that, compared with the TNF‐α group, the migration ability of HUVECs was significantly improved after 24 h of treatment with EVs, miRNA‐125b, and LET‐EVs‐miRNA‐125b. The scratched surface in the LET‐EVs‐miRNA‐125b group almost healed after 24 h (Figure 6D). The tube formation assay showed that LET‐EVs‐miRNA‐125b significantly promoted the angiogenesis of HUVECs (Figure 6E). These results showed that LET‐EVs‐miRNA‐125b had a good therapeutic effect on improving cell viability, inhibiting apoptosis, and promoting angiogenesis in vitro.

### 2.6 RNA‐Sequencing Analysis and Potential Mechanisms Underlying the Effects of LET‐EVs‐miRNA‐125b

**Figure 7:**
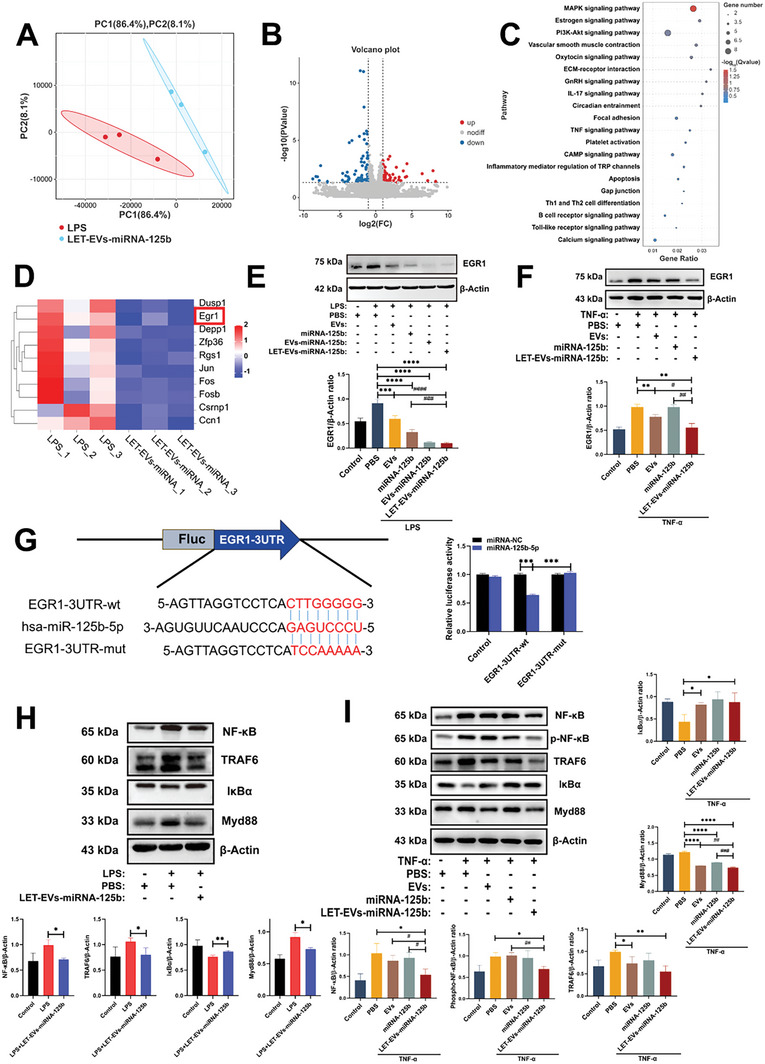
RNA‐sequencing analysis of lung tissues after LET‐EVs‐miRNA‐125b treatment. A) The principal component analysis between the LPS and LET‐EVs‐miRNA‐125b treatment groups. B) Volcano plot of Log2 fold‐changes in gene expression after LET‐EVs‐miRNA‐125b treatment (124 differentially expressed genes were identified, of which 54 were upregulated and 70 were downregulated). C) A large number of inflammation and endothelial barrier‐related pathways were enriched by KEGG pathway enrichment analysis. D) Top 10 differentially expressed genes between the LPS and LET‐EVs‐miRNA‐125b treatment groups. E,F) The expression of EGR1 in lung tissues and HUVECs in different groups. The levels of EGR1 were quantified using ImageJ and normalized to those of β‐Actin. G) The potential binding site between miRNA‐125b‐5p and EGR1 was validated using a dual‐luciferase reporter assay. Data are presented as the mean ± SD (*n* = 3). ^***^ *p* < 0.001. H,I) The expressions of NF‐κB inflammatory pathway proteins in lung tissues and endothelial cells, normalized to that of β‐Actin. Data are presented as the mean ± SD (*n* = 3). ^*^ *p* < 0.05, ^**^ *p* < 0.01, ^****^ *p* < 0.0001, compared with the PBS treatment group by unpaired Student's *t*‐tests. ^#^ *p* < 0.05, ^##^ *p* < 0.01, ^###^ *p* < 0.001, compared with the LET‐EVs‐miRNA‐125b treatment group by unpaired Student's *t*‐tests.

To explore the potential mechanism by which LET‐EVs‐miRNA‐125b repaired the endothelial barrier in ALI, we conducted RNA sequencing analysis of lung tissues from the control, LPS, and LET‐EVs‐miRNA‐125b groups. The results revealed 1518 differentially expressed genes between the control and LPS groups, of which 1069 were upregulated and 449 were downregulated (Figure S9A,B, Supporting Information). Pathway enrichment analysis was performed using the kyoto encyclopedia of genes and genomes (KEGG) database. Many inflammatory and innate immune‐related pathways were enriched, indicating that the inflammatory response cascade is crucial in ALI development (Figure S9C, Supporting Information). In order to compare the difference between LET‐EVs‐miRNA‐125b group and LPS group, a principal component analysis (PCA) and variance analysis were carried out. The results showed good reproducibility of samples within groups and significant differences between samples across groups (**Figure** **7**A). After LET‐EVs‐miRNA‐125b was administered, 124 differentially expressed genes were detected, of which 54 were upregulated and 70 were downregulated compared to those in the LPS group (Figure 7B). KEGG pathway enrichment analysis showed a large number of inflammation and endothelial barrier‐related pathways, which suggested that LET‐EVs‐miRNA‐125b was involved in inflammation and endothelial barrier regulation in ALI (Figure 7C). Among the top 10 differentially expressed genes, we identified an important transcription factor, EGR1, which is involved in the regulation of inflammation and angiogenesis in endothelial cells (Figure 7D). At the protein level, we verified that EGR1 expression was upregulated after LPS stimulation and downregulated after treatment with LET‐EVs in lung tissues (Figure 7E). In addition, we found that in the LET‐EVs‐miRNA‐125b group, the levels of the NF‐κB signaling markers TRAF6, NF‐κB, and MYD88 were decreased (Figure 7H). The expressions of EGR1 and NF‐κB signaling pathway‐related proteins were further validated in endothelial cell injury models at the protein level, and the results showed the same trend (Figure 7F,I). miRNAs can downregulate gene expression at the transcriptional and post‐transcriptional levels.^[^ ^20^ ^]^ We found that miRNA‐125b‐5p could bind to EGR1 mRNA 3′‐untranslated regions (3′‐UTR) by sequence alignment. A dual‐luciferase reporter assay verified this targeting relationship (Figure 7G). In conclusion, LET‐EVs‐miRNA‐125b inhibits the expression of EGR1 by delivering miRNA‐125b to damaged endothelial cells, thereby regulating inflammation and endothelial barrier repair.

## 3 Discussion and Conclusion

ALI and ARDS are multifactorial diseases. At present, there is a lack of effective treatment for ARDS. Novel non‐cellular therapies based on EVs have provided new insights into the treatment of ALI.^[^ ^4^, ^8^ ^]^ Currently, MSC‐derived EVs are considered effective alternatives to cell therapy. In ALI, MSC‐EVs can reduce pulmonary inflammation and protect the air‐blood barrier by preserving the structure of alveolar epithelial cells and microvascular endothelial cells, polarizing anti‐inflammatory macrophages, and inhibiting neutrophil infiltration and neutrophil extracellular traps formation.^[^ ^4^ ^]^ However, MSC‐EVs treatment still faces some limitations in clinical settings, such as limited cell sources, the high doses required for administration,^[^ ^21^ ^]^ unstable content fraction,^[^ ^10^ ^]^ and tumorigenicity.^[^ ^15^ ^]^ Endothelial cell‐derived EVs (EC‐EVs) are promising alternatives. Compared to MSCs, endothelial cells provide a viable, abundant, and readily available source of EVs. Under physiological conditions, EC‐EVs encapsulate specific proteins (e.g., VE‐cadherin and angiotensin‐converting enzyme) and nucleic acid components (e.g., miR‐126, miR‐25, and miR‐221) that protect the endothelial barrier and promote angiogenesis.^[^ ^22^ ^]^ Several studies have reported that EC‐EVs can effectively reduce myocardial ischemia‐reperfusion,^[^ ^23^ ^]^ improve ischemic stroke,^[^ ^24^ ^]^ prevent atherosclerosis,^[^ ^25^ ^]^ and exert significant therapeutic effects in various endothelial injury diseases. In the present study, treatment with EC‐EVs alleviated lung injury and repaired the endothelial barrier in mice to some extent^[^ ^1^, ^26^ ^]^; however, the efficacy needed to be improved (Figure 3).

Native EVs have poor lung tissue targeting abilities and can only carry low amounts of therapeutic substances, which are the most significant problems in EV therapy.^[^ ^27^ ^]^ Tracing of DIR‐labeled EVs in vivo showed that, after intravenous administration, large amounts of EVs accumulated in the liver and spleen. Only a small number of EVs arrived at the lung tissues, and they only remained there for a short time.^[^ ^11^ ^]^ Therefore, EVs cannot effectively act on damaged lung tissues. In addition, miRNAs that play a major regulatory role are present at low levels in native EVs, therefore they cannot effectively exert therapeutic effects.^[^ ^28^ ^]^ To solve these problems and improve therapeutic efficiency, we modified EC‐EVs to establish eEVs.

miRNAs are small non‐coding RNAs. In ALI/ARDS, miRNAs regulate inflammatory and immune responses and alleviate lung injury.^[^ ^29^ ^]^ MiRNA‐125b is an important small RNA involved in the regulation of ALI.^[^ ^30^ ^]^ A previous study reported that miRNA‐125b‐5p is highly expressed in endothelial cells and plays an important protective role in endothelial injury. Under oxidative stress conditions, overexpression of miRNA‐125b‐5p can protect endothelial cells and reduce apoptosis and necrosis.^[^ ^31^ ^]^ In various ALI models, upregulating the expression of miRNA‐125b maintained weight and survival, reduced lung inflammation, and reversed endothelial barrier damage in mice.^[^ ^18^, ^32^ ^]^ The upregulation of miRNA‐125b levels in EVs has an important protective effect against ALI. Furthermore, Shen et al. reported that miR‐125b‐5p in MSC‐EVs can attenuate ferroptosis in HPMVECs due to excessive inflammation in ALI by regulating the expression of Keap1/NRF2/GPX4.^[^ ^33^ ^]^ Therefore, miRNA‐125b was chosen to be loaded into the EVs. EC‐EVs serving as nano vectors loaded with miRNA‐125b not only promoted miRNA delivery in vivo but also exhibited improved therapeutic efficiency, achieving a “two birds with one stone” effect.

In this study, LET was obtained by using phage display technology to screen out immortalized lung microvascular endothelial cells. To evaluate their targeting ability in vivo, biotin‐labeled LET peptide was injected into the mice via the tail vein. The results showed that significant biotin signals were expressed in lung tissues and were co‐localized with CD31. When the LET peptide linked to D(KLAKLAK)2 pro‐apoptotic factor was administered to C57 mice, the alveolar structure was damaged, but there were no significant changes in kidney, heart, liver, or bone marrow tissues. Thus, the LET peptide had an effective ability to target lung endothelial cells, therefore LET was chosen to modify the surface of EVs.^[^ ^17^, ^34^ ^]^ Our study showed that LET‐EVs exhibited superior lung targeting and accumulation abilities. After 48 h, a large number of LET‐EVs remained in the lung tissues. Compared with EVs, LET‐EVs can play a role in lung tissues that improve the therapeutic effect in ALI by ameliorating lung injury and reducing inflammation. In addition, because of the lung endothelial ability, LET‐EVs could directly act on HPMVECs in vivo and decreased vascular permeability which played an important role in repairing endothelial barrier integrity. The results showed that LET‐EVs had better anti‐inflammatory and barrier repair effects compared to the EVs group. However, LET‐EVs could not achieve the ideal therapeutic efficacy, so it was necessary to modify the LET‐EVs to construct eEVs‐LET‐EVs‐miRNA125b (Figure 2; Figure S6, Supporting Information).

In this study, an endothelium‐derived engineered EV (LET‐EVs‐miRNA‐125b) was proposed for the first time and its efficacy was evaluated in LPS‐induced ALI mice. The results showed that, compared to that of the EVs, miRNA‐125b, and EVs‐miRNA125b treatment groups, the LET‐EVs‐miRNA‐125b treatment group had better therapeutic effects in ALI, relieving lung tissue lesions, maintaining the original alveolar structure, reducing inflammatory cell infiltration, and protecting the air‐blood barrier (Figures 3 and 4; Figure S5, Supporting Information).

We then further examined the protective effect of the constructed LET‐EVs‐miRNA‐125b on endothelial barrier integrity in ALI. The endothelial barrier is an important component of the air‐blood barrier, which is usually the first component to be damaged in ALI and determines the development and prognosis of ALI.^[^ ^35^ ^]^ Abnormal apoptosis of HPMVECs and disruption of intercellular junctions are the main causes of disruption of endothelial barrier integrity. Therefore, the inhibition of endothelial cell apoptosis, promotion of angiogenesis, and protection of intercellular junctions are important for protecting endothelial barrier integrity.^[^ ^4^ ^]^ LET‐EVs‐miRNA‐125b effectively regulates these three components and plays an important protective role in endothelial barrier integrity. LET‐EVs‐miRNA‐125b could effectively inhibit endothelial cell apoptosis. TUNEL fluorescence staining of the lung tissues showed that the number of apoptotic cells in the lung tissues was significantly reduced by LET‐EVs‐miRNA‐125b treatment (Figure 3G). In the endothelial injury cell model, administration of LET‐EVs‐miRNA‐125b reduced the apoptotic rate of endothelial cells (Figure 6B). Furthermore, LET‐EVs‐miRNA‐125b promoted angiogenesis. The results showed that LET‐EVs‐miRNA‐125b promoted the proliferation, migration, and angiogenesis of injured endothelial cells within 24 h in the endothelial cell injury model, exerting a significant pro‐repair effect on injured endothelial cells (Figure 6C–E). Endothelial intercellular junctions are important regulatory factors that determine the endothelial barrier integrity.^[^ ^36^ ^]^ The pulmonary microvascular endothelial barrier is mainly composed of tight junction proteins (ZO‐1, Occludin, and claudin) and adhesion junction proteins (VE‐cadherin), which are distributed at the junctions of adjacent endothelial cells.^[^ ^36^ ^]^ In this study, LET‐EVs‐miRNA‐125b can restore intercellular junctions. After LET‐EVs‐miRNA125b treatment, vascular permeability and TEER values of the endothelial barrier were significantly recovered. IF and IB analysis of ZO‐1, Occludin, and VE‐cadherin expression in lung tissues and endothelial cells showed that their expression was significantly increased in the LET‐EVs‐miRNA‐125b group. Overall, LET‐EVs‐miRNA‐125b played an important role in protecting the endothelial barrier integrity (Figure 5; Figure S7, Supporting Information).

**Figure 8:**
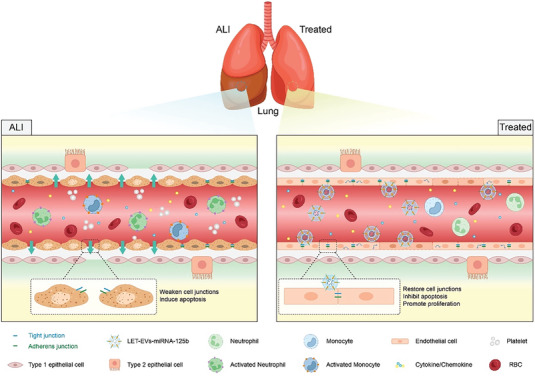
Mechanism diagram. During the development of ALI, large amounts of inflammatory factors and neutrophils infiltrate cells and the pulmonary air‐blood barrier becomes disrupted. Many endothelial cells become apoptotic and intercellular junctions are disrupted in ALI. Thus, the integrity of the endothelial barrier is destroyed. LET‐EVs‐miRNA‐125b has a significant therapeutic effect on ALI by reducing the inflammatory response and protecting the air‐blood barrier. LET‐EVs‐miRNA‐125b can target and deliver miRNA‐125b to endothelial cells. The expression of EGR1 in the endothelium is downregulated, which restores cell junctions, inhibits apoptosis, and promotes proliferation.

To explore the potential mechanism by which LET‐EVs‐miRNA‐125b repaired the endothelial barrier in ALI, comparative RNA‐sequencing analysis of lung tissues between the LPS and LET‐EVs‐miRNA‐125b groups was conducted. Out of the top 10 differentially expressed genes between the LPS and LET‐EVs‐miRNA‐125b groups, we focused on the transcription factor EGR1, which is associated with inflammation and the endothelial barrier. EGR1 belongs to the EGR family of Cys2His2‐type zinc‐finger proteins,^[^ ^37^ ^]^ which can be activated by various stimuli, including growth factors, cytokines, radiation, injury, and mechanical stress.^[^ ^38^ ^]^ EGR1 is an important transcription factor that mediates inflammation and barrier damage in endothelial cells by regulating multiple signaling pathways. EGR1 is also involved in inflammation and the systemic coagulation cascade response by activating the RhoA/ROCK/YAP signaling pathway.^[^ ^39^ ^]^ Activation of Gas/cAMP/PKA signaling inhibits the nuclear accumulation of EGR1, which promotes angiogenesis and vascular remodeling.^[^ ^40^ ^]^ Therefore, inhibiting the expression of EGR1 is an effective approach for endothelial injury treatment. In the ALI lung tissues and endothelial cell injury model, we proved that LET‐EVs‐miRNA‐125b could downregulate the expression of EGR1 at the protein level. LET‐EVs‐miRNA‐125b also upregulates the expression of junction proteins and downregulates the expression of NF‐κB signaling pathway‐related proteins. A dual‐luciferase reporter assay revealed that miRNA‐125b‐5p targeted EGR1(Figure 7). Therefore, we inferred that LET‐EVs‐miRNA‐125b inhibits the expression of EGR1 by delivering miRNA‐125b to damaged endothelial cells, thereby regulating inflammation and endothelial barrier repair (**Figure** **8**).

LET‐EVs‐miRNA‐125b has shown excellent therapeutic efficacy in preclinical studies but its clinical application has some limitations. Here, LET was modified onto the surface of EVs by lentiviral transfection, which carries the risk of genomic integration in clinical application. Therefore, safer modification methods such as transient transfection and chemical ligation will be used for subsequent clinical studies. Another limitation of our study is the lack of research on other cells in lung tissue. The pathogenesis of ALI is complex, with various cells involved in the development of the disease. In this study, we focused on the effects of the eEVs on repairing the endothelial barrier but did not investigate its effects on other cell types. The changes in different cell populations in the alveolar space will be explored in future studies. Moreover, the potential mechanism underlying the downregulation of EGR1 by LET‐EVs‐miRNA‐125b needs to be further explored.

In the present study, we successfully designed and constructed endothelium‐derived eEVs (LET‐EVs‐miRNA‐125b) for the first time. Our study demonstrated that LET‐EVs‐miRNA‐125b was remarkably effective in treating ALI compared to EVs and miRNA‐125b. LET‐EVs‐miRNA‐125b was delivered to HPMVECs to protect the integrity of the endothelial barrier. The delivery of miRNA‐125b in endothelial cells is a potential mechanism of the action of LET‐EVs‐miRNA‐125b.

## 4 Experimental Section

### Cell Culture

HUVECs and HPMVECs were acquired from ScienCell (San Diego, California, USA). HPMVECs were cultured in a complete cell medium supplemented with 1% endothelial cell growth (Sciencell, San Diego, California). HUVECs were cultured in complete Dulbecco's Modified Eagle's medium (DMEM; BasalMedia, Shanghai, China). All cells were cultured at 37 °C and 5% CO_2_.

### Plasmid Construction and Preparation of Lentivirus

The plasmid was provided by Echobiotech (Beijing, China). Briefly, lung endothelial cell‐targeted peptides (LET and CGSPGWVRC) and LAMP‐2B were synthesized using PCR, with cDNA as a template. The LAMP‐2B‐LET gene was then recombined into the pLVX‐Puro blank plasmid using Xhol and BamHI restriction enzyme sites. The lentivirus was generated by transient transfection of HEK293T cells using the LAMP‐2B‐LET expressing vector. HUVECs seeded in a 6‐well plate were transfected with the LAMP‐2B‐LET lentivirus for 24 h. The supernatant containing the lentivirus was discarded and replaced with a fresh complete culture medium to continue the culture. After 72 h, puromycin (Yeason, Shanghai, China) was used to extract the transfected HUVECs.

### EV Isolation

The serum‐free cell culture medium (200 mL) was collected. The culture medium was sequentially subjected to centrifugation to remove cells, cell debris, and apoptotic bodies at 4 °C. Amicon centrifugal filters (100 kDa MWCO, UFC9100; Millipore Sigma, St Louis, Missouri, USA) were used to concentrate the supernatant to 2 mL. Sepharose CL‐2B (Solarbio, Beijing, China) was used as the filler and PBS (Servicebio, Wuhan, China) was used as the flow phase. The columns (φ17 mm; Titan, Shanghai, China) were incubated at 4 °C and washed with PBS. A concentrated supernatant (1 mL) was loaded onto the column, and the eluent was labeled fraction 1. Twenty‐four fractions (2 mL per fraction) were collected as indicated. EV‐enriched fractions (fractions 6–10) were collected and further concentrated using Amicon centrifugal filters (10 kDa MWCO, UFC9010; Millipore, St Louis, Missouri, USA) to a final volume. EVs were stored at −80 °C until the next experiment.

### Preparation of LET‐EVs‐miRNA‐125b

The plasmid expressing the LAMP‐2B‐LET gene was packaged into a lentivirus. The LAMP‐2B‐LET lentivirus was added into the culture medium of HUVECs to induce the cells to stably express the LAMP2B‐LET gene. The transfected cells were cultured and the supernatants were collected. LET‐EVs were extracted and isolated by size exclusion. For loading and purification, LET‐EVs were mixed with 20 pmol (in vitro experiment) or 200 pmol (in vivo experiment) miRNA125b (AS02F6FP; Ambion, USA) and 10 µL of Exo‐Fect Exosome transfection reagent (SBI, San Francisco, California), then incubated for 10 min at 37 °C. ExoQuick (SBI, San Francisco, California) was added to LET‐EVs, and they were incubated for 30 min at 4 °C. Then, the mixtures were centrifuged for 3 min at 13 000–14 000 rpm in a microfuge. The supernatant was removed and the precipitant was resuspended in 100 µL PBS. LET‐EVs‐miRNA‐125b was stored at −80 °C until the next experiment.

### NTA

The particle and size of EVs were detected by nanoparticle tracking analysis at VivaCell Shanghai, using a ZetaView PMX 110 (Particle Metrix, Meerbusch, Germany) and the corresponding software, ZetaView 8.04.02 SP2.

### TEM

EVs were added to Formvar carbon‐coated grids and left to adsorb (<1 min), followed by negative staining with a phosphotungstic acid solution for 1–10 min. The filter paper was then used to absorb the liquid, and the grid was air‐dried. Images were acquired using a biological transmission electron microscope (FEI TECNAI G2 12).

### Western Blot

The total protein from lung tissues, cells, and EVs was extracted using RIPA lysis buffer (Epizyme, Shanghai, China). The concentration was measured using a Bradford Protein Assay Kit (Beyotime, Shanghai, China), as per the manufacturer's instructions. Proteins were isolated using a sodium dodecyl sulfate‐polyacrylamide gel (SDS‐PAGE; Epizyme, Shanghai, China) and then transferred onto a polyvinylidene difluoride membrane (PVDF, Millipore Sigma, St. Louis, Missouri, USA). The membrane was blocked using 3% BSA (Amresco, Washington) for 2 h at room temperature and then incubated with primary antibody overnight at 4 °C. Primary antibodies specific for LAMP2B (A0593; Abclonal, Wuhan, China), CD63 (AF5117; Affinity, Shanghai, China), CD81 (ab79559; Abcam, Cambridge, Britain), TSG101 (28283‐1‐AP; Proteintech, Chicago, USA), Calnexin (ab22595; Abcam, Cambridge, Britain), ZO‐1 (61‐7300; Invitrogen, Carlsbad, California), Occludin (40‐4700, Invitrogen, Carlsbad, California), VE‐cadherin (A12416; Abclonal, Wuhan, China), EGR1 (A2722; Abclonal, Wuhan, China), NF‐κB (4764S; CST, Boston, Massachusetts), p‐NFκB (3033S; CST, Boston, Massachusetts), IκBa (A19714; Abclonal, Wuhan, China), MYD88 (4283S; CST, Boston, Massachusetts), TRAF6 (Ag14858; Proteintech, Wuhan, China), β‐Actin (ab8226; Abcam, Cambridge, Britain), and GAPDH (ab181602; Abcam, Cambridge, Britain) were used in this study. After being washed with Tris‐buffered saline (TBS, Servicebio, Wuhan, China) containing 1% Tween‐20 (Bio‐light, Shanghai, China), the membrane was incubated with horseradish peroxidase‐labeled secondary antibody goat anti‐mouse IgG (H+L) (31 430, Thermo, USA) or goat anti‐rabbit lgG (H+L) (31 460, Invitrogen, Carlsbad, California) for 1 h at room temperature (RT). The membranes were washed to remove unbound antibodies, and a Chemiluminescent HRP Substrate (WBKLS0500; Millipore, St. Louis, Missouri) was added to detect protein bands. The membranes were scanned using a gel imager (BLT, GelView 6000Plus) and analyzed using ImageJ software.

### Quantitative Real‐Time (qRT)‐PCR

Total RNA was extracted from the lung tissues, cells, and EVs using RNAiso Plus (TAKARA, Japan). RNA was reverse transcribed into cDNA (Vazyme, Suzhou, China). For the reverse transcription of miRNAs, a polyA tailing method was conducted using the miRNA 1st Strand cDNA Synthesis Kit (Vazyme, Suzhou, China). The U6 small nuclear RNA (snRNA) was used for quantification. cDNA was detected by qPCR using the Vazyme Taq Pro Universal SYBR qPCR Master Mix (Vazyme, Suzhou, China). The relative expressions of mRNA and miRNA were calculated using the 2^−ΔΔCt^ method and normalized to GAPDH and U6, respectively. The primers for miRNA‐125b‐5p and U6 were purchased from RiboBio (Shanghai, China). The primers are shown in Table S1 (Supporting Information).

### Cellular Uptake

Purified EVs were incubated with 10 µm of the cell membrane dye DIL (Solarbio, Beijing, China) for 30 min. Unbound DIL dye was removed with three washes with PBS buffer (pH 7.4, 3000 g, 20 min per wash) in a 10 kDa MWCO Amicon centrifugal filter at 4 °C. HPMVECs were seeded in 24‐well plates. DIL‐labeled EVs or LET‐EVs were added into cells and incubated for 2 and 24 h at 37 °C after cells reached ≈70% confluency. Then, the cells were washed with PBS to remove EVs that were not taken up and were fixed with 4% paraformaldehyde (Servicebio, Beijing, China). F‐actin was stained with Alexa Fluor 488 phalloidin (Invitrogen, Carlsbad, California). Nuclei were stained with DAPI (Beyotime, Shanghai, China). Fluorescence microscopy was used to observe the uptake efficiency of LET‐EVs or EVs.

### Distribution of DIR‐Labeled EVs

Purified EVs were labeled with the cell membrane dye DIR (Delta, Szechwan, China). The NEWTON 7.0 imaging system (Vilber, France) was used to observe the biodistribution of DIR‐labeled EVs in mice. In vivo images were captured of ALI mice 2 and 48 h after intravenous administration. The mice were then sacrificed, and the major organs (lungs, brain, liver, heart, spleen, kidneys, and intestines) were collected for ex vivo imaging.

### Immunofluorescence

Formalin‐fixed, paraffin‐embedded (FFPE) blocks of lung tissue were removed from paraffin and dehydrated. Liquid nitrogen snap‐frozen lung tissues were dehydrated and fixed in 4% paraformaldehyde. EDTA Antigen Retrieval Solution (pH 6.0; Solarbio, Beijing, China) was used for antigen repair. Then, 3% hydrogen peroxide was used to block the activity of endogenous peroxidase. Sera was incubated with sections for 30 min to block nonspecific binding. The blocking solution was removed and diluted primary antibody solution containing CD31 (ab182981; Abcam, Cambridge, Britain), Ki‐67 (ab15580; Abcam, Cambridge, Britain), ZO‐1 (61‐7300; Invitrogen, Carlsbad, California), VE‐cadherin (ab33168; Abcam, Cambridge, Britain), and Occludin (40‐4700; Invitrogen, Carlsbad, California) was incubated with sections overnight at 4 °C. After being washed with PBS three times, the sections were incubated with secondary antibody for 1 h. The sections were then washed three times with PBS and shaken dry. PBST containing 0.003% hydrogen peroxide was incubated with sections for 30 min. Finally, DAPI was used to stain cell nuclei. Images were acquired using a scanner.

### Confocal Microscopy

HUVECs were seeded into a confocal dish with TNF‐α (H8916; Sigma, St Louis Missouri, USA) for 4 h, and then were treated with EVs, miRNA‐125b, and LET‐EVs‐miRNA‐125b for 24 h. HUVECs were fixed in pre‐cooled methanol (Titan, Shanghai, China) for 30 min and occluded with 3% BSA. The primary antibodies against ZO‐1 (61‐7300, Invitrogen, Carlsbad, California), VE‐cadherin (ab33168, Abcam, Cambridge, Britain), and Occludin (40‐4700, Invitrogen, Carlsbad, California) were incubated with cells overnight at 4 °C. Cells were washed three times and incubated with goat anti‐rabbit IgG H&L Alexa Fluor 488 (ab150077, Abcam, Cambridge, UK) or goat anti‐rabbit IgG H&L Alexa Fluor 555 (ab150078, Abcam, Cambridge, UK) for 1 h at RT. The nuclei were re‐stained with DAPI. Finally, a fluorescence confocal microscope (Zeiss LSM710 Meta, Carl Zeiss) was used to capture images and the fluorescence intensity was analyzed using ImageJ.

### Lipopolysaccharide‐Induced Acute Lung Injury Model

BALB/c and C57BL/6 mice (18–20 g, 6–8 weeks, wild‐type, male) were purchased from Sino‐British SIPPR/BK Lab Animal Ltd. (Shanghai, China). BALB/c mice were used for EVs in vivo tracking assays. C57BL/6 mice were used to establish the ALI animal model and evaluate the efficacy of LET‐EVs‐miRNA‐125b. First, 50 µL 1 mg kg^−1^ LPS (Sigma, St. Louis, Missouri, USA) was administered to mice by tracheal nebulization using a nebulizing needle from Bio Jane Trading Limited (Shanghai, China). After 4 h, PBS (200 µL), EVs (1 × 10^10^ particles in 200 µL), microRNA125b (200 pmol in 200 µL), EVs‐miRNA‐125b (1 × 10^10^ particles in 200 µL), and LET‐EVs‐miRNA‐125b (1 × 10^10^ particles in 200 µL) were intravenously administered to mice. After 48 h, mice were euthanized using an aerosolized isoflurane overdose, and lung tissues were obtained for lung injury evaluation.

### Total Protein and Cytokine Concentration in BALF

After euthanasia, 0.8 mL PBS was used to lavage the total collected lung tissue three times. BALF was collected in a centrifuge tube and centrifuged at 1500 g for 15 min to remove cells. Then, the supernatant was collected and stored at −80 °C. The concentrations of proteins and the cytokines (TNF‐α, IL‐6, and IL‐10) were determined using Bradford Protein Assay and Elisa kits (F11630, F10830, F10870; Westang, Shanghai, China).

### Wet to Dry Weight Ratio

The lung tissues were obtained and weighed as the wet weight. Then, they were transferred into an oven and dried to a constant weight at 72 °C, which was considered the dry weight. The wet weight was divided by the dry weight to obtain the final wet to dry weight ratio

### Histopathological Staining and Wright–Giemsa Staining

Lung tissues and BALF cells were fixed in 4% paraformaldehyde and embedded in paraffin. The paraffins were then cut into 4‐µm‐thick sections using paraffin slicer machines for H&E staining and Wright–Giemsa staining.

### Evans Blue

The mice were injected intravenously with 50 mg kg^−1^ Evans Blue solution (Sigma, St. Louis, Missouri, USA), which was left to circulate in vivo for 2 h. The mice were then euthanized and lung tissues were collected. Lung tissues were weighed, cut, and transferred to tubes. Formamide (Sinopharm, Beijing, China) was incubated with lung tissues in an oven to extract Evans Blue. The supernatant was obtained by centrifugation and the absorbance at 620 nm was measured using an enzyme meter.

### Transendothelial Electrical Resistance

HUVECs were seeded in Transwell filter inserts (6.5 mm, 3.0 µm; Corning Life Sciences, Lowell, MA, USA) and cultured for 7 days to form a cell monolayer. TEER was measured using a Voltohmmeter (Millicell ERS‐2; Millipore) to record the initial resistance. Then, HUVECs were pretreated with TNF‐α (10 ng mL^−1^) for 4 h and followed by PBS, EVs, miRNA‐125b, and LET‐EVs‐miRNA125b. TEER values were recorded after 24 h. Values were normalized to those of the control group.

### CCK‐8 Experiment

Cell viability was determined using a Cell Counting Kit‐8 assay (CCK‐8, VB563, Dojindo Laboratories, Japan). Briefly, HUVECs were seeded in 96‐well plates at 4 × 10^4^ cells cm^−2^ with TNF‐α for 4 h and then were treated with EVs, miRNA125b, and LET‐EVs‐miRNA125b. The supernatant was discarded, and the medium containing CCK‐8 solution was incubated for 1 h at 37 °C. The absorbance was measured at 450 nm using a microplate reader.

### Flow Cytometry

Apoptosis of HUVECs was evaluated using the Annexin V‐FITC/PI assay (40302ES50; Yeason, Shanghai, China). HUVECs were seeded in 6‐well plates, incubated with TNF‐α, and then treated with EVs, microRNA125b, and LET‐EVs‐microRNA125b. The cells were then digested and washed. The binding buffer was added to resuspend cells. Then, 5 µL Annexin V‐FITC and PI was added to cells and was incubated for 15 min. Flow cytometry was used to determine the apoptosis rate of HUVECs (Beckman CytoFlex).

### EDU

Cell proliferation was detected using EdU (Beyotime, Shanghai, China). The treatment of HUVECs was similar to that during the CCK‐8 assay. The medium was discarded and EdU solution was added for 2 h at 37 °C. HUVECs were fixed and made transparent using 0.3% Triton X‐100 (Beyotime, Shanghai, China). The EdU click reaction was then performed. HUVECs were added to 100 µL Click reaction solution, incubated for 30 min at RT in the dark, and washed and labeled with DAPI. A fluorescence microscope was used to capture images.

### Cell Migration Assay

HUVECs were seeded into 6‐well plates to cultivate overnight. When the cells reached full confluency as a monolayer, they were stimulated for 4 h. A pipette tip was used to create scratches in the monolayer. HUVECs were washed using PBS to remove the floating cells. EVs, miRNA125b, and LET‐EVs‐miRNA125b were added to 1% FBS complete medium and cultivated with cells for 24 h. Changes in the scratches were captured using an inverted electron microscope. ImageJ software was used to calculate the scratch area and the migration rate was calculated using the following formula:

(1) $$\begin{array}{ccc} \mathrm{Migration}\;\mathrm{rate}\;\left(\%\right) & = & (\mathrm{initial}\;\mathrm{wound}\;\mathrm{size}-\mathrm{wound}\;\mathrm{size}\;\mathrm{after}\;24\;\mathrm{hours})/\\ & & \left(\mathrm{initial}\;\mathrm{wound}\;\mathrm{size}\right)\times 100\% \end{array}$$

### Tube‐Formation Assay

A tube‐formation assay was used to evaluate the angiogenic capacity of HUVECs. Briefly, 90 µL Matrix gel (Corning, New York, NY, USA) was added to each well of a 96‐well plate. The 96‐well plate was then placed in the refrigerator at 4 °C for 10 min to flatten the liquid surface and then the well plate was transferred into an incubator for 40 min to solidify the Matrigel. HUVECs (1000 cells/well) were seeded into 96‐well plates containing the matrix gel and cultivated for 4 h. Subsequently, images were obtained using an inverted microscope, and the number of cell rings was counted using ImageJ software.

### RNA Sequencing

Total RNA was extracted from the lung tissues of the control, LPS, and LET‐EVs‐microRNA125b groups using RNAiso Plus. RNA sequencing and analysis were performed by Guangzhou Gene Denovo Biotechnology (China). The differentially expressed genes were confirmed with standards of *p* ≤ 0.05, |log2FC|≥1. Pathway enrichment analysis was performed using the KEGG database in OmicShare (https://www.omicshare.com/).

### Dual‐Luciferase Reporter Assay

A wild‐type EGR1 vector (EGR1‐3UTR‐wt) and a mutant 3ʹ‐UTR of EGR1 (EGR1‐3UTR‐mut) were constructed by Shanghai Zorin Biotechnology Co., Ltd. HUVECs were seeded in 24‐well plates and co‐transfected with miRNA‐125b‐5p/miRNA‐NC and luciferase vectors (EGR1‐3UTR‐wt/ EGR1‐3UTR‐mut) for 48 h. Luciferase activity was measured using the Dual‐Luciferase Reporter System Assay Kit (Promega, E1910).

### Statistical Analyses

All graphical and statistical analyses were performed using GraphPad Prism 8.0.1 and ImageJ. Quantitative evaluation of grayscale, fluorescence intensity, and migration area were performed using ImageJ software. All data were obtained from three independent parallel experiments, and the data are presented as the mean ± SD. Unpaired Student's *t*‐tests were used to compare data between two groups. *p* < 0.05 was considered statistically significant. (^*^ *p* < 0.05, ^**^ *p* < 0.01, ^***^ *p* < 0.001, ^****^ *p* < 0.0001). All the animal experiments were approved by the ethics committee on animal use at Naval Medical University. All procedures used in the experiments complied with the guidelines of the National Ethics Committee on Animal Welfare of China and were approved by the Ethics Committee on Animal Use of Naval Medical University (No. 20210310013).

## Conflict of Interest

The authors declare no conflict of interest.

## Supporting information

Supporting Information

## Data Availability

The data that support the findings of this study are available from the corresponding author upon reasonable request.
