# Supplementary material for: Endothelium‐Derived Engineered Extracellular Vesicles Protect the Pulmonary Endothelial Barrier in Acute Lung Injury
Source: Adv Sci (Weinh). 2023 Dec 7;11(6):2306156. doi: 10.1002/advs.202306156 (PMC10853733; doi:10.1002/advs.202306156)
Supplement: Supplementary file 1 — Supporting Information [file ADVS-11-2306156-s001.pdf]

ADVANCED SCIENCE
Open Access

# Supporting Information

for *Adv. Sci.*, DOI 10.1002/advs.202306156

Endothelium-Derived Engineered Extracellular Vesicles Protect the Pulmonary Endothelial Barrier in Acute Lung Injury

*Zhengyan Gu, Mingxue Sun, Jihao Liu, Qi Huang, Yunqin Wang, Jun Liao, Tingbin Shu, Min Tao, Guanchao Mao, Zhipeng Pei, Wenqi Meng, Xinkang Zhang, Youheng Wei, Shanshan Zhang, Songling Li, Kai Xiao*, Ying Lu* and Qingqiang Xu**

Supporting Information

# Endothelium-derived engineered extracellular vesicles protect the pulmonary endothelial barrier in acute lung injury

Zhengyan Gu[#1,2], Mingxue Sun[#1], Jihao Liu[#1], Qi Huang[3], Yunqin Wang[1], Jun Liao[2,4], Tingbin Shu[1], Min Tao[1], Guanchao Mao[1], Zhipeng Pei[1], Wenqi Meng[1], Xinkang Zhang[1], Youheng Wei[5], Shanshan Zhang[1], Songling Li[1], Kai Xiao[1,6*], Ying Lu[2*], Qingqiang Xu[1,7*]

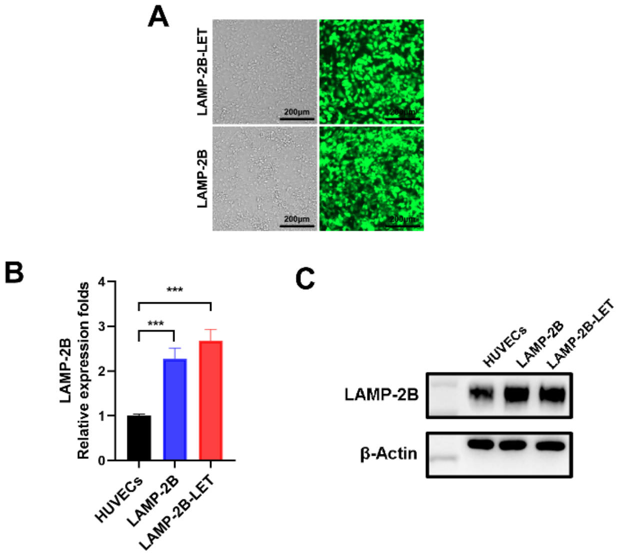


**Figure S1. Transfection efficiency of lentivirus in HUVECs.**
(A) Fluorescence microscopy images of HUVECs transfected with LAMP-2B and LAMP-2B-LET lentivirus and screened with puromycin (Scale bar: 200 μm). (B) Relative expression of LAMP-2B in HUVECs after transfection with LAMP-2B and LAMP-2B-LET lentivirus detected by qRT-PCR. Data were presented as Mean ± SD (n = 3). ****P* < 0.001, compared with HUVECs by unpaired Student's t-tests. (C) Protein expression of LAMP-2B in HUVECs after transfection with LAMP-2B and LAMP2B-LET lentivirus (n = 3).

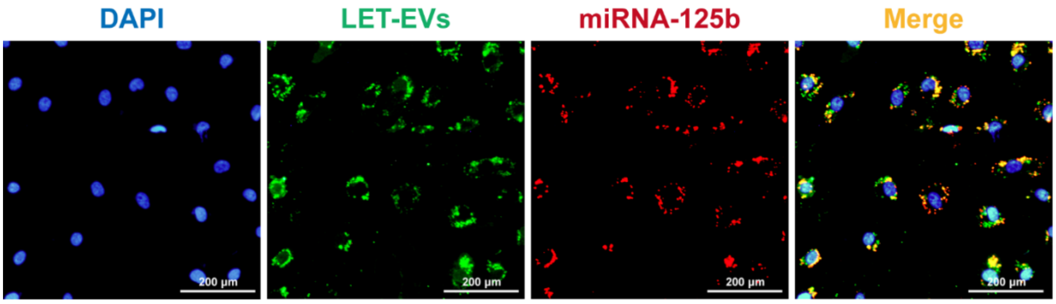


**Figure S2. Delivery of Cy5 labeled miRNA-125b to HUVECs by LET-EVs.** The Cy5 labeled miRNA-125b was loaded into DIO labeled LET-EVs. The labeled LET-EVs-miRNA-125b were co-cultured with HUVECs for 2 h. HUVECs were fixed with paraformaldehyde and stained with DAPI. The localization of Cy5 labeled miRNA125b and DIO labeled LET-EVs was observed by fluorescence microscopy, suggesting LET-EVs could deliver miRNA125b into HUVECs. Stains used are as follows: DIO labeled EVs (Green), Cy5 labeled miRNA125b (Red), and DAPI (blue) (Scale bar: 200 μm).

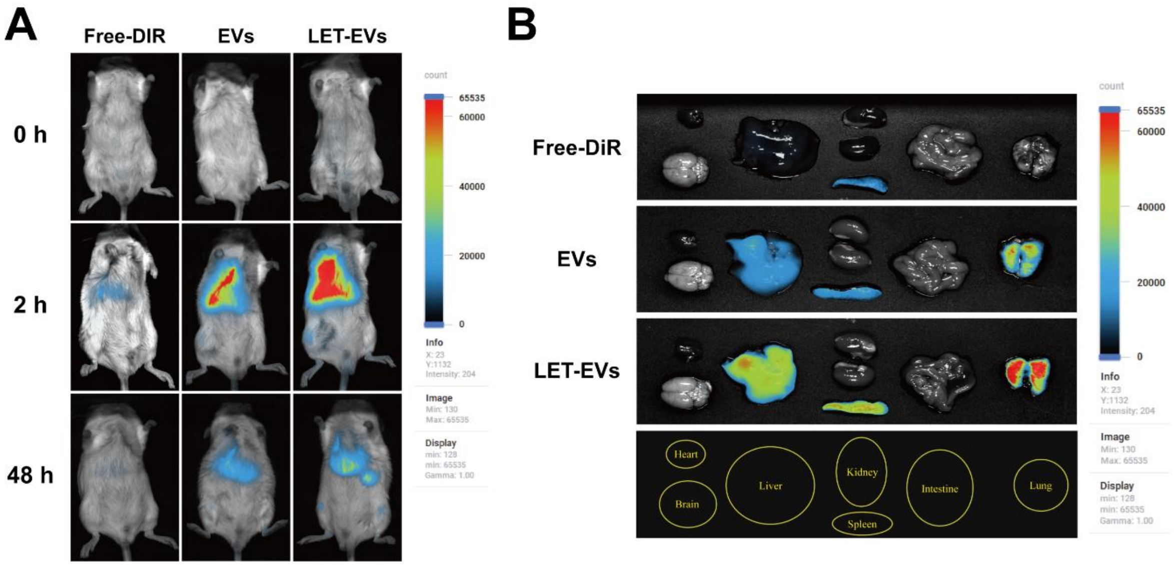


**Figure S3. Distribution of DIR labeled LET-EVs and EVs in healthy mice.**
(A) Imaging of healthy mice after 0, 2 and 48 h of administration of DIR, DIR-EVs and DIR -LET-EVs. Compared with DIR and DIR-EVs group, the fluorescence signal of LET-EVs mainly accumulated in epigastric after 2 h. (B) Fluorescence imaging of organs of healthy mice in DIR, DIR-EVs and DIR-LET-EVs group after 48 h. Compared with DIR and DIR-EVs group, signal in DIR-LET-EVs groups mainly gathered in lung tissues.

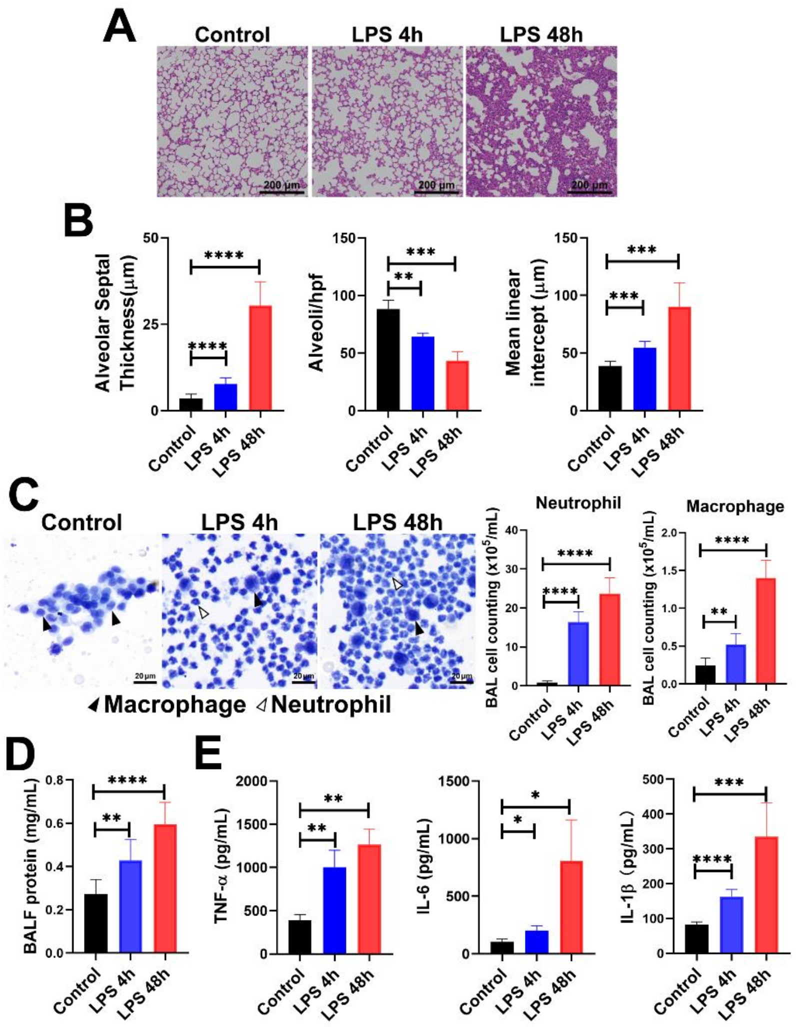


**Figure S4 Establishment and evaluation of acute lung injury animal models**

(A) Representative images of H&E-stained lung tissue sections after LPS stimulation for 4h and 48h(Scale bar: 200 μm). (B) The morphology evaluation of the lung tissues by reducing the number of alveoli/hpf and increasing the alveolar septal thickness and the mean linear intercept after LPS after LPS stimulated for 4 h and 48 h in compared with the control group. (C) Wright-Giemsa staining and cell count of inflammatory cell. A large number of neutrophils were aggregated in the alveoli/interstitium after giving LPS.(D) The protein concentration in BALF was increased in LPS treatment group compared to the control group.(E) The concentration of TNF-α, IL-6, and IL-1β in BALF was increased after LPS stimulation. Data were presented as Mean ± SD (n = 3). **P* < 0.05, ***P* < 0.01, ****P* < 0.001, *****P* < 0.0001, compared with control group by unpaired Student's t-tests.

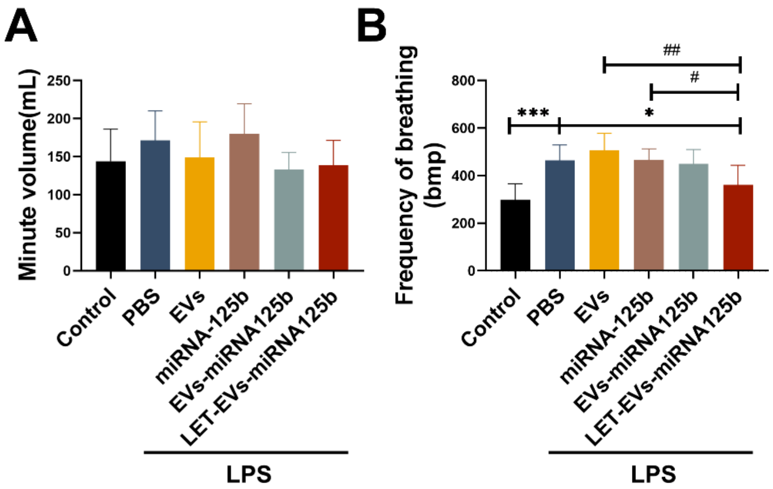


**Figure S5 .Standard pulmonary function test in ALI mice**

(A) Minute volume and (B) Frequency of breathing in different groups detected by the small animal respiratory physiological detection system of EMKA Company. All the data were presented as Mean ± SD (n = 3). **P* < 0.05, ****P* < 0.001, compared with PBS treatment group by unpaired Student's t-tests. $^{\#}P < 0.05$, $^{\#\#}P < 0.01$, compared with LET-EVs-miRNA-125b treatment group by unpaired Student's t-tests.

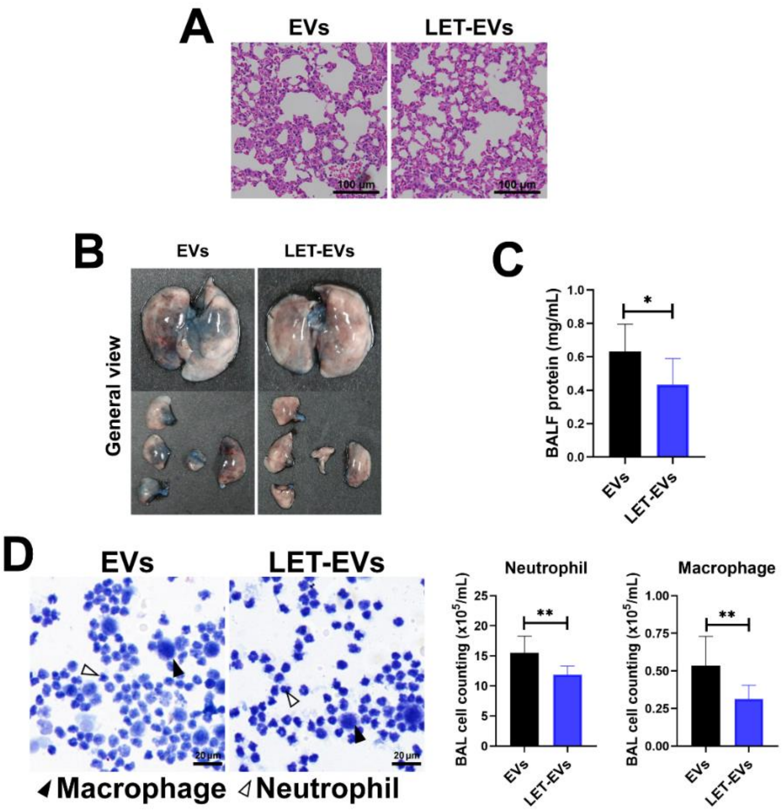


**Fig S6. Comparison of the efficacy between EVs and LET-EVs.** (A) Representative images of H&E-stained lung tissue sections between EVs and LET-EVs group(Scale bar: 100 μm). (B) Representative images of lung tissues after staining with Evans blue. (C) Protein concentrations in BALF in EVs group compared to those of the LET-EVs group. (D) Wright-Giemsa staining and cell count of inflammatory cells (Scale bar: 100 μm).

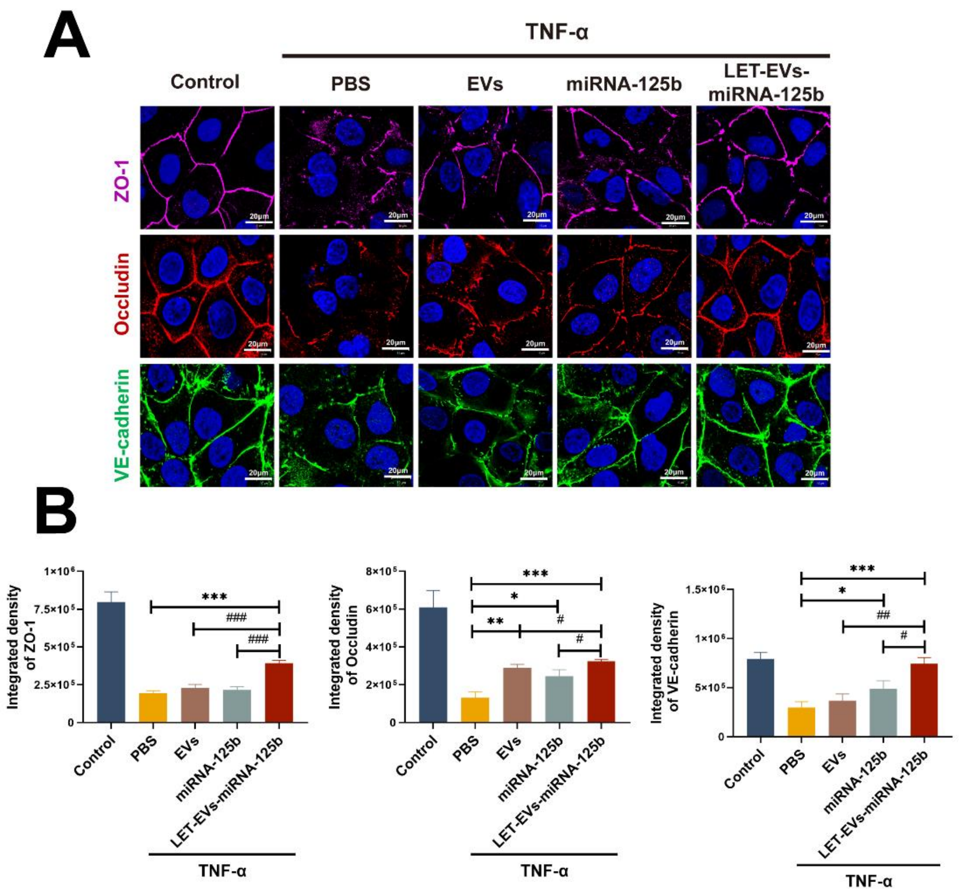


**Figure S7. The distribution of junction proteins in the endothelial cell model.**
(A)ZO-1 (Magent), Occludin (Red), and VE-cadherin (Green) immunofluorescent staining results of different treatments of endothelial cells. Nuclei were stained with DAPI (Blue) (Scale bar: 20 μm).(B) Quantification of ZO-1, Occludin and VE-cadherin fluorescence integrated density based on ImageJ analysis. Results represent the mean ± SD (n = 3). *$P$ < 0.05, **$P$ < 0.01, ***$P$ < 0.001, compared with PBS treatment group by unpaired Student's t-tests. $^{\#}P$ < 0.05, $^{\#\#}P$ < 0.01, $^{\#\#\#}P$ < 0.001, compared with LET-EVs-miRNA-125b treatment group by unpaired Student's t-tests.

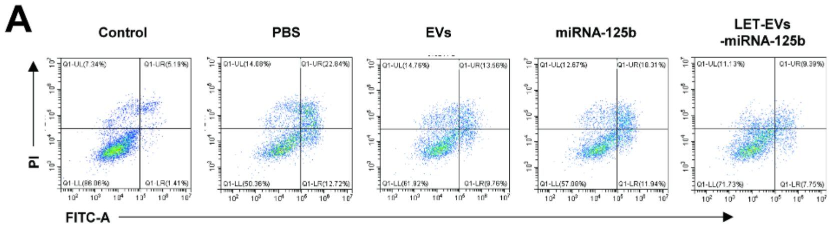


**Figure S8. The rate of apoptosis in HUVECs was determined using Annexin V/PI flow cytometry.**

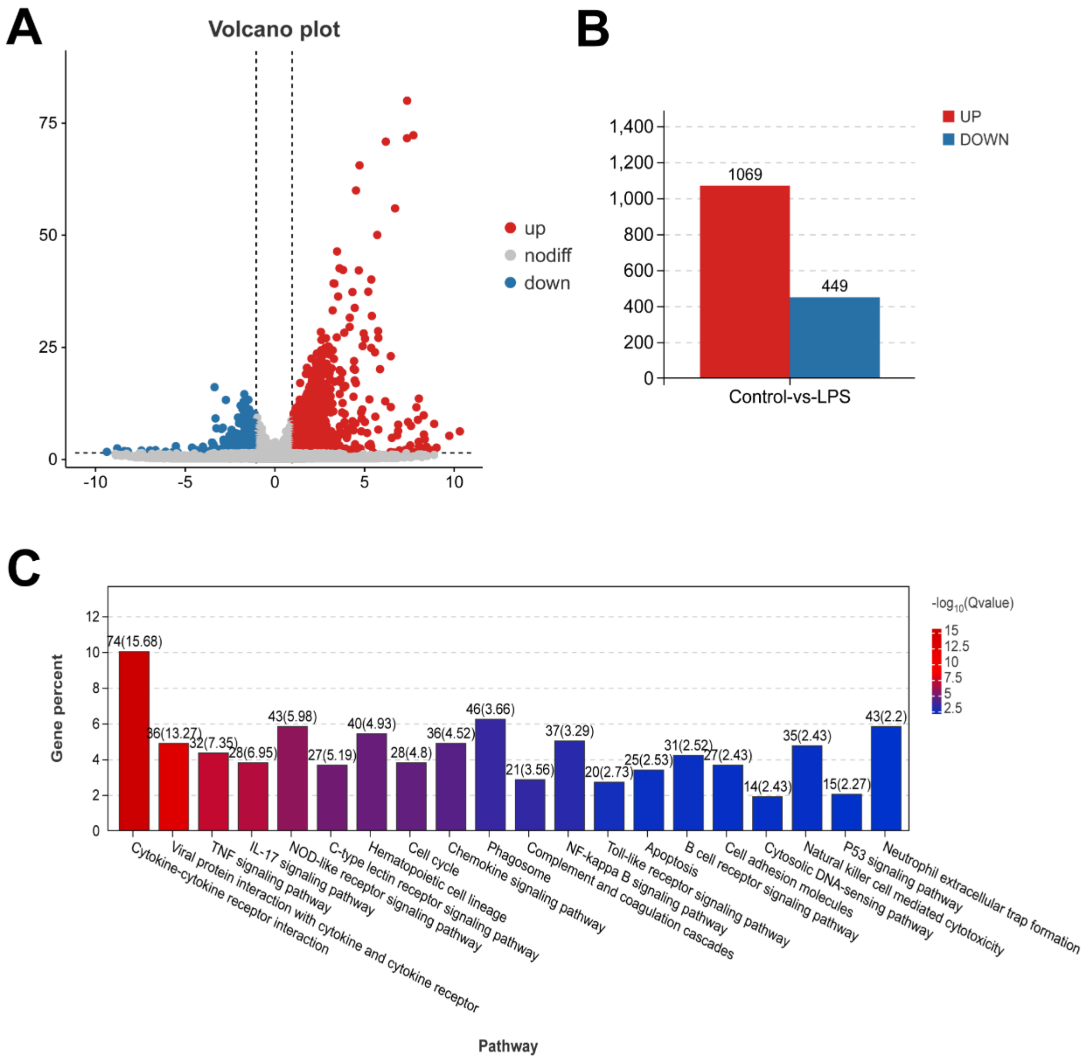


**Figure S9. RNA-sequencing analysis of lung tissues in the control and LPS groups.**
(A) Volcano plot of Log2 fold-change in gene expression between the control and LPS groups. (B) Histogram of genes with altered expression. (1518 differentially expressed genes, of which 1069 were up-regulated and 449 were down-regulated) (C) KEGG pathway enrichment analysis.

**Table 1 Primer sequence**

| Gene | Primer sequence (5′–3′) |
| --- | --- |
| Human LAMP-2B | Forward: TGGTGTGCTTCCGCCTCTTC |
| | Reverse：TGCATAAAGGCAAGTGGC |
| Human GAPDH | Forward：AACATCTACAAGCCCAACAACAAGG |
| | Reverse：GGTTCTGCAATCACATCTTCAAAGTC |

**Table 2 Lung injury scoring system**

| Parameter | Score per field | | |
|---|---|---|---|
| | 0 | 1 | 2 |
| A. Neutrophils in the alveolar space | none | 1-5 | >5 |
| B. Neutrophils in the interstitial space | none | 1-5 | >5 |
| C. Hyaline membranes | none | 1 | >1 |
| D. Proteinaceous debris filling the airspaces | none | 1 | >1 |
| E. Alveolar septal thickening | <2× | 2×-4× | >4× |

Score=[(20×A) + (14×B) + (7×C) + (7×D) + (2×E)] / (number of fields×100)

The "Pathological lung injury score" was performed by a pathologist who was unaware of the study group. The pathologist scored pathological damage on H&E-stained lung tissue sections, with 20 randomly selected 400× high magnification fields of view in each group, in which alveoli should occupy at least 50% of each field of view and areas consisting mainly of lumens of large airways or blood vessels should be excluded. Pathologists assigned values to five independent variables: neutrophils in the alveolar space, neutrophils in the interstitial space, hyaline membranes, proteinaceous debris filling the airspaces, and alveolar septal thickening and weighted them to obtain the final lung injury score.
